# HEV prevalence and potential risk factors in a large multi-ethnic youth cohort in China

**DOI:** 10.1186/s12985-020-01470-3

**Published:** 2021-01-06

**Authors:** Huixia Li, Yinxia Zhang, Zhongren Ma, Zewen Liu, Aqsa Ikram, Lijiang Liu, Guoqin Zhao, Qiuwei Pan, Zulqarnain Baloch

**Affiliations:** 1 Key Laboratory of Biotechnology and Bioengineering of State Ethnic Affairs Commission, Biomedical Research Center, Northwest Minzu University, Lanzhou, China; 2 Faculty of Life Science and Technology, Kunming University of Science and Technology, Kunming, 650500 Yunnan China

**Keywords:** Hepatitis E virus, Immunoglobulin M, Immunoglobulin G, Ethnic, China

## Abstract

**Background:**

This cohort study was designed to investigate the prevalence of and potential risk factors of HEV infection in a large multi-ethnic youth cohort in China.

**Methods:**

Blood samples were collected from participants (n = 6269) and serum was isolated. All serum samples were tested for anti-HEV IgG, anti-HEV IgM antibodies using commercial enzyme immunoassay kits (Wantai Biological Pharmacy Enterprise, Beijing, China).

**Results:**

The overall rate of anti-HEV IgG and anti-HEV IgM prevalence was 4.78% and 0.14%, 0.03% were positive for both anti-HEV IgG and anti-HEV IgM antibodies. Anti-HEV IgG positivity is significantly higher in females (5.27%) compared to males (4.14%) (*P = *0.028). Anti-HEV IgG prevalence is significantly (*P* = 0.0001) higher in Dong (17.57%), Miao (12.23%), Yi (11.04%), Gelao (9.76%), and Bai (10.00%) compared to other ethnic groups. It is significantly higher in Guizhou (11.4%), Sichuan (10.1%), Yunnan (9.3%), and Guangxi (6.9%) than that other province. We found that ethnicity and provincial background are significantly associated with HEV infection in this cohort.

**Conclusion:**

This study provides comprehensive information on HEV prevalence in multi-ethnic populations in China. However, our study only focused on a youth population from different provinces of China. Future studies are recommended to investigate HEV prevalence in other age groups of the ethnic populations.

## Background

Hepatitis E, caused by the Hepatitis E virus (HEV) has emerged as an important health concern in both developed and developing countries [1]. In general, HEV infection is asymptomatic and self-limiting [2, 3], but it can also cause severe diseases in specific populations, including pregnant women, immunocompromised patients, and patients with underlying liver diseases [4–6]. Globally, it is responsible for approximately 20 million new cases, over 3 million acute hepatitis cases, and 70,000 fatalities annually [7, 8]. The HEV outbreaks were mainly common in developing countries compared to developed countries and its prevalence significantly varies based on ethnicity, socioeconomic conditions, food habits, water quality, sanitation and, geographic origin [9, 10].

The People’s Republic of China is the leading populated country in the world, is comprised of 56 different ethnic groups with different dietary habits and living lifestyle. Therefore, different Chinese populations are prone to many food-borne infectious diseases, such as HEV infection. According to recent studies, the overall sero-prevalence of anti-HEV immunoglobulin G (IgG) among the general Chinese people’s ranges from 11–72% [7, 9, 11], and that of IgM is 1.8% [9]. However, HEV prevalence among different ethnic populations at the national level has not been comprehensively studied. The majority of the ethnic populations are living in less developed areas in isolated communities with relatively poor sanitation, unhygienic food practices, socioeconomic condition, and lack of health facilities for prevention, diagnosis, and treatment of infectious diseases. Therefore, we designed this cohort study to investigate the sero-prevalence of IgG and IgM in large multi-ethnic Chinese youth and to understand potential risk factors.

## Methods

### Study design

We conducted this large multi-ethnic youth cohort study among the freshly enrolled students of Northwest Minzu University, a university dedicating to high education for ethnic populations in China. 5 ml of blood was taken in sterile syringes from 6269 students and was immediately transported to the laboratory for further processing. In addition to sample collection, we used structured questionnaire forms to document participants’ demographic characteristics such as age, sex, ethnicity, area, political status, major subject, college, City, province. The serum samples were stored at −80 °C, until further testing. In this study, all participants were above 16 > year’s age and never get HEV vaccination before.

### Serological tests

Serum samples were tested for the presence of anti-HEV IgG and IgM antibodies using commercial-available enzyme immunoassay kits (Wantai Biological Pharmacy Enterprise, Beijing, China) according to the manufacturer’s instructions. Samples were tested in duplicate with cutoff values for IgG and IgM assays set at 0.22 and 0.357, respectively, which were determined based on the mean optical density 450 values from the negative controls (6 standard deviations). Samples with OD greater than or equal to the cutoff value were considered as positive.

### Statistical analysis

The data was analyzed using SPSS version 20.0 (Chicago, IL, USA). Proportions were estimated with the 95% confidence interval (CI). *χ*^*2*^ test or Fisher’s exact test and Mantel-extension test for trend were performed to evaluate the difference in the prevalence of viral markers among sex, age, residential, and occupational groups. Univariate analysis using the *χ*^*2*^ test or Fisher’s exact test and multivariate logistic regression analysis was performed to identify potential risk factors for HEV infection by calculating odds ratios (ORs) and 95% CI. For all analyses, *P*-value < 0.05 was considered statistically significant.

## Results

### Demographic characteristics of the participants

The demographic characteristics of all participants are shown in Table 1. In total, there were 6269 students, comprising of 3589 female and 2680 male. Age distribution was from 16 > to 31 years (mean, 18.7 ± 1.25; median, 19 years). Among 6269 students, 1502 belonged to rural and 4678 to urban areas. A total of 36.56% of students were Han, 13.49% were Hui, 8.85% were Tibetan, 5.89% were Zhuang, 5.63% were Tujia, 4.47% were Uighur, 4.43% were Miao, 3.91% were Mongol, and the remaining belonged to others ethnicities (Table 1). In this study, 14.15% of students belong to Gansu province followed by Guizhou (8.23%), Guangxi (6.91%), Xinjiang (6.81%), Qinghai (6.52%), Yunnan (6.36%), Ningxia (5.44%), Inner Mongolia (4.64%), Hunan (4.47%), Sichuan (4.10%), Tibet (3.68%), Chongqing (3.56%), and the remaining were from others provinces (Additional file 1: Appendix Table 1).

**Table 1:** Demographic characteristics of participants

Ethnic	Total	Percentage
Sex		
Female	3589	57.25
Male	2680	42.75
Ethnic		
Bai	60	0.96
Bouyei	74	1.18
Dong	74	1.18
Dongxiang	124	1.98
Gelao	41	0.65
Han	2292	36.56
Hui	846	13.49
Kazakh	40	0.64
Li	69	1.10
Man	118	1.88
Miao	278	4.43
Mongol	245	3.91
Monguor	54	0.86
She	26	0.40
Tibetan	555	8.85
Tujia	353	5.63
Uighur	280	4.47
Yao	56	0.89
Yi	154	2.46
Zhuang	369	5.89
Others*	161	2.98
Age		
≤ 18 years	3069	48.96
19 & 20 years	2859	45.61
≥ 21 years	341	5.44
Area		
Rural	1502	23.96
Urban	4678	74.62

*Other ethnics (Dai: 5, Daur:4, Derung:1, Hani:7, Hezhen:1, AcHang: 1, Blang: 2, Bonan: 3, Evenki:1, Gin: 2, Lahu: 1, Kirgiz/Kirghiz: 8, Korean:7, Lisu:10, Maonan: 2, Mulam: 10, Nakhi/Naxi: 12, Nu:1, Pumi: 3, Qiang: 3, Russ:1, Salar: 16, Sui: 13, unknown, 31, Uzbek:1, Va: 2, Xibe: 6, Yugur: 7)

### HEV sero-prevalence

**Fig. 1:**
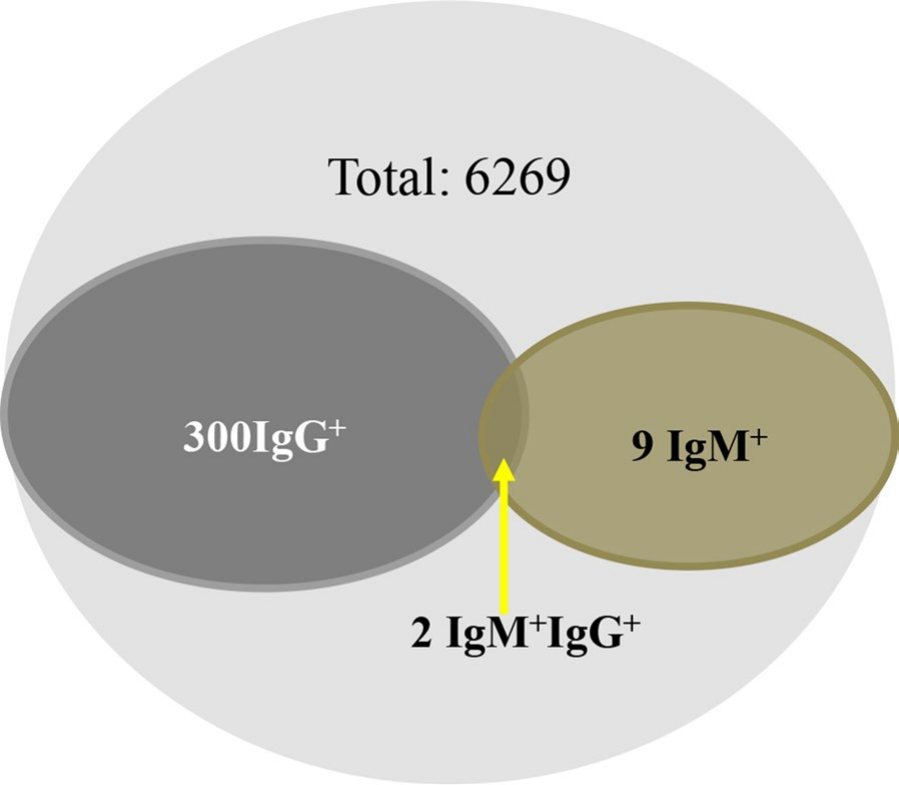
Anti-HEV IgG and IgM antibodies Sero-prevalence in the Young Chinese population

The overall sero-prevalence of anti-HEV IgG and IgM antibodies positive was 4.96% (311/6269). Among them, 300 participants (4.79%) were anti-HEV IgG antibody positive IgM antibody negative, indicating past HEV infection. 11 participants (0.18%) were positive with anti-HEV IgM, among them 2 participants (0.03%) were positive for both anti-HEV IgG and IgM and 9 were negative for anti-HEV IgG, indicating recent/ongoing infection (Fig. 1). The results of serologic testing are shown in Table 2. Overall anti-HEV IgG (Past infection) prevalence was 4.79% (300/6269; [95% CI: 4.27–5.34]) and anti–HEV IgM (Recent or ongoing infection) prevalence was 0.18% [95%CI: 0.09–0.31]. We next performed analysis on the characteristics of Anti-HEV IgG positivity (past infection) because IgM or both IgG and IgM positive samples are very limited. Anti-HEV IgG positivity was significantly higher in female participants (5.27%) compared to male participants (4.14%) (*P = *0.028). Similarly, anti-HEV IgG prevalence was significantly (*P* = 0.0001) higher in Dong (17.57%), Miao (12.23%), Yi (11.04%), Bai (10.00), and Gelao (9.76%) than that of other ethnic groups (Table 2).

**Table 2:** HEV prevalence among various groups

Ethnic	Total	Anti-HEV IgG positive (rate %)	*P* value	Anti-HEV IgM positive (rate %)	*P*-value
**Overall**	6269	300 (4.8)		11(0.18)	
**Sex**			0.028*		0.37
Female	3589	189 (5.3)		8 (0.20)	
Male	2680	111 (4.1)		3 (0.11)	
**Ethnic**			0.0001*		
** Bai**	**60**	**6 (10.00)**		**0**	
** Bouyei**	**74**	**6 (8.1)**		**0**	
Dong	74	**13 (17.6)**		0	
Dongxiang	124	2 (1.6)			
** Gelao**	**41**	**4 (9.8)**		**0**	
Han	2292	92 (4.0)		3 (0.13)	
Hui	846	16 (1.9)		2 (0.24)	
Kazakh	40	0 (0.00)		0	
Li	69	3 (4.3)		1 (1.45)	
Man	118	2 (1.7)		0	
** Miao**	**278**	**34 (12.2)**		**2 (0.72)**	
Mongol	245	4 (1.6)		0	
Monguor	54	4 (7.4)		0	
She	26	1 (3.8)		0	
** Others**	**161**	**13 (8.1) **		**0**	
Tibetan	555	31 (5.6)		0	
Tujia	353	15 (4.2)		1 (0.28)	
Uighur	280	9 (3.2)		0	
** Yao**	**56**	**5 (8.9)**		**1 (0.35)**	
** Yi**	**154**	**17 (11.0)**		**1 (0.65)**	
Zhuang	369	23 (6.2)		0	
**Age**			0.645		0.29
≤18 years	3069	145 (4.7)		4 (0.13)	
19 and 20 years	2859	135 (4.7)		5 (0.17)	
≥21years	341	20 (5.9)		2 (0.59)	
**Area**			0.215		0.25
Rural	1502	82 (5.5)		4 (0.27)	
Urban	4678	216 (4.6)		7 (0.15)	

Bold values indicated statistically significant

Data were presented as n (%)

*The results from the *Chi Square Test*

**Fig. 2:**
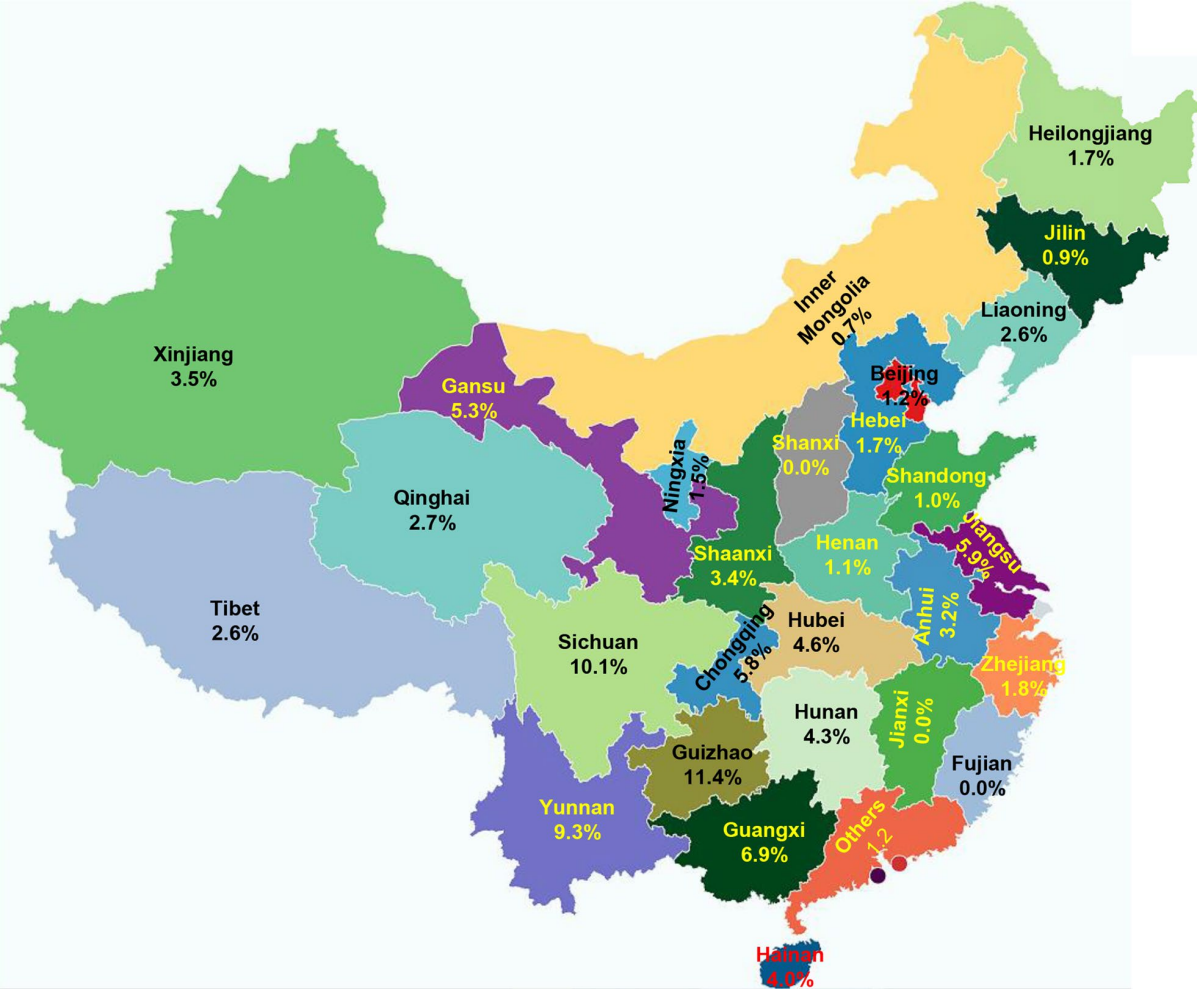
Map of Anti-HEV IgG prevalence in different provinces of China

Anti-HEV IgG prevalence was significantly different among participants from different provinces (*P* < 0.0001) (Fig. 2). The overall anti-HEV IgG rate was significantly higher in Guizhou (11.4%), Sichuan (10.1%), Yunnan (9.3%), and Guangxi (6.9%) than that of other provinces (Fig. 2). 11 IgM positive samples were tested for viral RNA by qRT-PCR, but HEV RNA was undetectable.

### Potential risk factors for HEV infection

To investigate the potential risk factors, only the samples positive for anti-HEV IgG were analyzed, which indicates past infection. Samples positive for IgM or both IgG and IgM, which indicates recent/ongoing infection, were not included because of the limited number. Univariate analysis revealed that sex, ethnic, and provincial backgrounds were significantly associated with a high rate of anti-HEV IgG antibody positivity. Interestingly, the multivariate analysis also indicated that ethnic and provincial background remained independent risk factors for the high rate of anti-HEV IgG positivity. The odds ratios and P-values of potential risk factors for anti-HEV IgG in the multivariate logistic regression model are shown in Table 3. In multivariate analysis, anti-HEV IgG positivity in females was higher compared to males but the difference was not significant (*P* = 0.058). Additionally, we found that there was a strong statistical association between geographic location and HEV prevalence (Table 3). Interestingly, in multivariate logistic ethnicity was also significantly (*P* = 0.017) associated with anti-HEV IgG positivity. The rate of anti-HEV IgG positivity was much higher in Dong, Miao, Yi, Gelao, Bai, and Yao compared to other ethnic groups (Table 3).

**Table 3:** Multivariate logistic regression analysis

Variables	Total	Positive for anti-HEV IgG (rate %)	*P*-value	OR (95% CI)
Sex			0.058*	
Female	3589	189 (5.3)		1
Male	2680	111 (4.1)		0.78 (0.62–1.00)
Ethnic			0.017*	
Overall	6269	300 (4.8)		1
Bai	60	6 (10.0)		1.67 (0.65–4.29)
Bouyei	74	6 (8.1)		1.33 (0.52–3.38)
Dong	74	13 (17.6)		3.21 (1.54–6.67)
Dongxiang	124	2 (1.6)		0.25 (0.06–1.06)
Gelao	41	4 (9.8)		1.63 (0.53–4.96)
Han	2292	92 (4.0)		0.63 (0.39–1.01)
Hui	846	16 (1.9)		0.29 (0.15–0.56)
Kazakh	40	0 (0.0)		0.0
Li	69	3 (4.3)		0.68 (0.20–2.34)
Man	118	2 (1.7)		0.26 (0.06–1.12)
Miao	278	34 (12.2)		2.1 (1.20–3.65)
Mongol	245	4 (1.6)		0.25 (0.08–0.73)
Monguor	54	4 (7.4)		1.20 (0.40–3.62)
Others	161	13 (8.1)		1.32 (0.40–3.62)
She	26	1 (3.85)		0.60 (0.08–4.64)
Tibetan	555	31 (5.6)		0.89 (0.51–1.55)
Tujia	353	15 (4.2)		0.67 (0.34–1.30)
Uighur	280	9 (3.2)		0.50 (0.23–1.10)
Yao	56	5 (8.9)		1.47 (0.54–4.05)
Yi	154	17 (11.0)		1.87 (0.97–3.60)
Zhuang	369	23 (6.2)		1.00 (0.65–1.48)
Province			0.00*	
Overall	6269	300 (4.8)		1
Anhui	62	2 (3.2)		0.97 (0.16–8.75)
Chongqing	223	13 (5.8)		1.85 (0.41–8.46)
Fujian	67	0 (0.0)		0
Gansu	887	47 (5.3)		1.68 (0.40–7.08)
Guangxi	433	29 (6.9)		2.23 (0.52–9.59)
Guizhou	516	59 (11.4)		3.87 (0.92–16.26)
Hainan	101	4 (4.0)		1.24 (0.22–6.96)
Hebei	118	2 (1.7)		0.52 (0.07–3.76)
Heilongjiang	119	2 (1.7)		0.51 (0.07–3.73)
Henan	176	2 (1.1)		0.34 (0.05–2.49)
Hubei	151	7 (4.6)		1.47 (0.30–7.27)
Hunan	280	12 (4.3)		1.34 (0.29–6.17)
Inner Mongolia	291	2 (0.7)		0.21 (0.03–1.50)
Jiangsu	51	3 (5.9)		1.87 (0.30–11.68)
Jiangxi	54	3 (5.6)		1.76 (0.28–10.98)
Jilin	112	1 (0.9)		0.27 (0.02–3.04)
Liaoning	152	4 (2.6)		0.81 (0.14–4.54)
Ningxia	341	5 (1.5)		0.45 (0.08–2.35)
Qinghai	409	11 (2.7)		0.83 (0.18–3.83)
Shaanxi	118	4 (3.4)		1.05 (0.19–5.91)
Shandong	104	1 (1.0)		0.29 (0.03–3.28)
Shanxi	51	0 (0.0)		0.00
Sichuan	257	26 (10.1)		3.38 (0.78–14.63)
Others	82	1 (1.2)		0.37 (0.03–4.18)
Tibet	231	6 (2.6)		0.80 (0.16–4.06)
Xinjiang	427	15 (3.5)		1.09 (0.24–4.89)
Yunnan	399	37 (9.3)		3.06 (0.72–13.06)
Zhejiang	57	1 (1.8)		0.54 (0.05–6.07)

*The ORs and 95% confidence intervals (CIs) use multivariate logistic regression analysis

^#^Others provinces (Beijing: 4, Guangdong: 18, Shanghai: 6, Tianjin: 6 and unknown (not mentioned provincial background detail: 48)

## Discussion

HEV prevalence in general populations varies extensively across the world [12]. Previous studies on HEV sero-prevalence in China indicate varying results based on ethnicity [10] and geographical location [11, 13]. It has been documented that Hani population has the highest prevalence (82.3%) followed by Naxi (71.9%), Bulang (65.1%),Wa (60.0%) [10], Han (22.28%), Mongolian (10.37%), Tibetan (17.99%), Uighur (46.61%), Zhuang (72.02%) and Hui (21.15%) [11]. Different from these segmented previous studies, we now comprehensively investigated HEV prevalence in a large Chinese cohort comprising of 47 ethnicities from 31 provinces. Among 6269 tested samples, the sero-prevalence of anti-HEV IgG was 4.96% and anti-HEV IgM was 0.18% (Fig. 1). In China, previous studies reported anti-HEV IgG sero-prevalence ranging from 10.8 to 66.58% in the general population [9, 10, 13], which is considerably higher as compared to our study. Similarly, anti-HEV IgM prevalence in this study was 0.14%, which is again lower as compared to previous studies, 0.84–1.8% among the general population [9, 10] and 0.5–5% in blood donors in China [14]. The potential reasons may be attributed to factors such as participants from diverse geographies, different populations, age groups [13, 15, 16], and the use of diagnostic assays with different sensitivities [17].

In this study, we found a high HEV prevalence in Dong (17.6%), Miao (12.23%), Yi (11.04%), Bai (10.2%), and Gelao (9.8%) than that of other ethnic groups (Table 2). The mmajority of Dong, Miao, Yi, Gelao, and Bai ethnic groups are populated in Sichuan, Yunnan, Guangxi, and Guizhou province of China. These provinces are mostly overpopulated and less developed [13, 18]. The anti-HEV IgG rate among the 28 provinces investigated in this study ranges from 0% to 11.4% (Fig. 2). The overall rate was higher in Guizhou (11.4%), Sichuan (10.1%), Yunnan (9.3%), and Guangxi (6.9%), consistent with our findings of high prevalence in ethnic groups mainly from these provinces [18].

When we analyzed different potential risk factors data acquired from the self-reported questionnaires using univariate and multivariate logistic regression analysis. Our analysis revealed that ethnicity and provincial background are significantly associated with anti-HEV IgG (Table 3). We found that anti-HEV IgG sero-prevalence was higher in females as compared to males but the difference was not significant. Similar results have been reported [19–21] but others observed the opposite [22, 23]. However, there is to date no evidence-based explanation on the sex disparity to HEV susceptibility, requiring further investigation. It is well accepted that HEV infection occurs at all ages, and thus the anti-HEV IgG rate increases with the growing of age. In this study, anti-HEV IgG prevalence ranges from 4.72–5.87% without significant differences among different age groups. This can be explained that we enrolled in a relatively homogeneous youth group. 94.6% of participants are less than 20 years old in this study, and therefore, young age might be the main contributor to the overall low HEV prevalence.

This study has limitations that must be considered when interpreting our findings. First, HEV RNA was not detected and therefore we could not confirm the circulating genotype in our population. Secondly, the information of several known risk factors, including dietary habits, living standards, travel history, family history, and profession were not available in this study. Finally, we were only able to recruit a youth cohort for practical reasons. Thus, the epidemiology of HEV infection in other age groups of Chinese ethnic populations remains to be investigated.

## Conclusion

This study provides comprehensive epidemiological information on HEV prevalence in the multi-ethnic populations residing in different provinces of China. We report the positive rate of anti-HEV IgG antibodies of 4.79% and IgM antibodies of 0.14%. HEV infection appears to be associated with sex, ethnic, and provincial background in our population.

## Supplementary information

**Additional file 1.** Participants provincial background.

## Data Availability

The aggregate data supporting findings contained within this manuscript will be shared upon request submitted to the corresponding author. Identifying patient data will not be shared.

## Abbreviation

HEV: Hepatitis E virus
