# Supplementary material for: HEV prevalence and potential risk factors in a large multi-ethnic youth cohort in China
Source: Virol J. 2021 Jan 6;18:3. doi: 10.1186/s12985-020-01470-3 (PMC7789705; doi:10.1186/s12985-020-01470-3)
Supplement: Supplementary file 1 — Additional file 1. Participants provincial background. [file 12985_2020_1470_MOESM1_ESM.docx]

**Appendix table 1**: Participants provincial background

| **Province** | **Numbers** |
| --- | --- |
| **Anhui** | 62 |
| **Chongqing** | 223 |
| **Fujian** | 67 |
| **Gansu** | 887 |
| **Guangxi** | 433 |
| **Guizhou** | 516 |
| **Hainan** | 101 |
| **Hebei** | 118 |
| **Heilongjiang** | 119 |
| **Henan** | 176 |
| **Hubei** | 151 |
| **Hunan** | 280 |
| **Inner Mongolia** | 291 |
| **Jiangsu** | 51 |
| **Jiangxi** | 54 |
| **Jilin** | 112 |
| **Liaoning** | 152 |
| **Ningxia** | 341 |
| **Qinghai** | 409 |
| **Shaanxi** | 118 |
| **Shandong** | 104 |
| **Shanxi** | 51 |
| **Sichuan** | 257 |
| **Tibet** | 231 |
| **Xinjiang** | 427 |
| **Yunnan** | 399 |
| **Zhejiang** | 57 |
| **Others** | 82 |
